# Itching Eruptions in Babies

**Published:** 1909-01-09

**Authors:** 


### Dermatology.

### ITCHING ERUPTIONS IN BABIES.

Until a baby is some months old it does not begin to use its fingers and nails for scratching. The presence of itching is until then indicated by restlessness, especially at night, and by the rubbing of the head and face upon objects within reach, and of the legs against one another. A pruritic eruption in an infant commonly belongs to one of the following groups; Eczema, lichen urticatus, scabies, or varicella. Eczema in an infant almost invariably occupies the face, and particularly the cheeks and forehead. In more severe cases it extends to the scalp, and patches may also appear upon the wrists and calves. It is seldom at this age very extensive upon the trunk and limbs, or, at any rate, it is patchy and not generalised. An infant with a generalised eczematous eruption, involving the trunk, limbs, and face uniformly, is almost always the victim of scabies. It is only in very young infants, however, that this generalised eczematous eruption is produced by scabies, and in babies of some months and older the eruption consists of scratched papules, small vesicles, pustules, and impetiginous crusts. The diagnosis rests then between scabies and lichen urticatus, and sometimes it is no easy matter to determine which of these affections it is. If the mother or the nurse has also an itchy eruption one may strongly suspect scabies; but the only certain test is in the finding of the burrows and the acarus. In babies the burrows are often found more readily upon the sides of the feet than on the hands. If, on the other hand, the case be one of lichen urticatus, careful search will generally reveal one or more of the papules or vesicles with an urticarial halo, and there will be a history of the occurrence of wheals in large numbers at night-time. There is a type of lichen urticatus which is sometimes really difficult to distinguish from varicella, and which is, in fact, often diagnosed as chicken-pox until it is found that the lesions still persist some weeks later. In the type of case referred to the characteristic wheals of lichen urticatus are centred, not by a fine papule, but by a clear vesicle, and when, during the day-time, the wheal subsides the clear vesicle remains. The difficulty of diagnosis is increased by the fact that the vesicle of varicella is often surrounded by an erythematous halo, and that it frequently itches considerably. The diagnosis can usually be made by attention to the following points: In varicella the lesions are generally present upon the face and scalp, and sometimes on mucous membranes, and usually there will be found some with a black crust; the lesions of lichen urticatus are prone to appear rather about the forearms and lumbar region, and to avoid the scalp and face; they are not seen upon mucous membranes, and they do not form black crusts.

